# Comparative genomics of freshwater Fe-oxidizing bacteria: implications for physiology, ecology, and systematics

**DOI:** 10.3389/fmicb.2013.00254

**Published:** 2013-09-12

**Authors:** David Emerson, Erin K. Field, Olga Chertkov, Karen W. Davenport, Lynne Goodwin, Christine Munk, Matt Nolan, Tanja Woyke

**Affiliations:** ^1^Bigelow Laboratory for Ocean Sciences, East Boothbay HarborME, USA; ^2^Los Alamos National Laboratory, DOE Joint Genome InstituteLos Alamos, NM, USA; ^3^DOE Joint Genome InstituteWalnut Creek, CA, USA

**Keywords:** Fe-oxidizing bacteria, *Sideroxydans*, Gallionellaceae, *Gallionella*, iron oxidizing bacteria

## Abstract

The two microaerophilic, Fe-oxidizing bacteria (FeOB) *Sideroxydans* ES-1 and *Gallionella* ES-2 have single circular chromosomes of 3.00 and 3.16 Mb that encode 3049 and 3006 genes, respectively. Multi-locus sequence analysis (MLSA) confirmed the relationship of these two organisms to one another, and indicated they may form a novel order, the Gallionellalaes, within the Betaproteobacteria. Both are adapted for chemolithoautotropy, including pathways for CO_2_-fixation, and electron transport pathways adapted for growth at low O_2_-levels, an important adaptation for growing on Fe(II). Both genomes contain Mto-genes implicated in iron-oxidation, as well as other genes that could be involved in Fe-oxidation. Nearly 10% of their genomes are devoted to environmental sensing, signal transduction, and chemotaxis, consistent with their requirement for growing in narrow redox gradients of Fe(II) and O_2_. There are important differences as well. *Sideroxydans* ES-1 is more metabolically flexible, and can utilize reduced S-compounds, including thiosulfate, for lithotrophic growth. It has a suite of genes for nitrogen fixation. *Gallionella* ES-2 contains additional gene clusters for exopolysaccharide production, and has more capacity to resist heavy metals. Both strains contain genes for hemerythrins and globins, but ES-1 has an especially high numbers of these genes that may be involved in oxygen homeostasis, or storage. The two strains share homology with the marine FeOB *Mariprofundus ferrooxydans* PV-1 in CO_2_ fixation genes, and respiratory genes. In addition, ES-1 shares a suite of 20 potentially redox active genes with PV-1, as well as a large prophage. Combined these genetic, morphological, and physiological differences indicate that these are two novel species, *Sideroxydans lithotrophicus* ES-1^T^ (ATCC 700298^T^; JCM 14762; DSMZ 22444; NCMA B100), and *Gallionella capsiferriformans* ES-2^T^ (ATCC 700299^T^; JCM 14763; DSMZ 22445; NCMA B101).

## Introduction

The freshwater Fe-oxidizing bacteria (FeOB) are a group of microbes associated with either natural or technical aqueous environments that contain appreciable concentrations of Fe(II). Because iron is the fourth most abundant element in the Earth's crust, these types of habitats are common and widespread; however due to the rapid chemical oxidation of Fe(II) in fully oxygenated waters they are restricted to redox boundaries where FeOB can flourish in opposing gradients of Fe(II) and O_2_ (Emerson et al., 2010; Hedrich et al., 2011). Despite historical recognition of the importance of FeOB in mediating Fe-oxidation (Harder, 1919), our detailed knowledge about the physiology and ecology of the FeOB is limited, in part due to the challenge of isolating pure cultures and developing model organisms from the few isolates available.

A number of recent reports have analyzed freshwater communities of FeOB using cultivation-independent approaches, and shown a dominant clade of operational taxonomic units (OTUs) in these communities belong to the Betaproteobacteria (Sahl et al., 2008; Duckworth et al., 2009; Wang et al., 2009; Bruun et al., 2010; Gault et al., 2011; Lin et al., 2011; Johnson et al., 2012). This clade includes the iconic, stalk-forming, *Gallionella ferruginea*, and other isolates, including *Sideroxydans* spp. of obligately microaerophilic FeOB (Weiss et al., 2007; Ludecke et al., 2010; Krepski et al., 2012). While prevalent in Fe-rich waters, members of this group are rarely, if ever, reported from other redox stratified habitats. These two lines of evidence suggest members of this lineage of Betaproteobacteria are uniquely adapted for growth on Fe(II) as an energy source.

Genomic analysis has proven useful in the analysis of acidophilic FeOB. Detailed individual analysis of genomes from strains of the well-studied *Acidithiobacillus ferrooxidans*, as well as comparative genomics studies, have yielded important insights into how this organism can conserve energy from Fe(II) oxidation for chemolithoautotrophic growth (Valdés et al., 2008). *Leptospirillum ferrooxidans*, and related species, play a crucial role in Fe-oxidation in acid mine drainage. Metagenomic analysis of communities dominated by *Leptospirillum* spp. have served as a model for understanding the population biology of naturally occurring microbial communities, and revealed the importance of specific cytochromes for Fe-oxidation (Tyson et al., 2004; Jeans et al., 2008; Singer et al., 2008).

This paper focuses on knowledge gleaned from genomics of two isolates of freshwater, neutrophilic FeOB, *Gallionella* strain ES-2, and *Sideroxydans* strain ES-1 (Emerson and Moyer, 1997). These two organisms were isolated from ferrugineous groundwater in Michigan, and described as obligate, microaerobic Fe-oxidizers. The genomic information presented here further develops our understanding of the physiology of these two organisms, and better delineates their respective ecological niches. This analysis also elucidates the taxonomy of these two organisms and the systematics of FeOB, and provides clues as to the mechanism of how they might conserve energy from the oxidation of iron.

## Materials and methods

### DNA isolation and genome sequencing

*Sideroxydans* ES-1, and *Gallionella* ES-2, were grown in the laboratory using standard conditions for the growth of neutrophilic, microaerobic FeOB (Emerson and Floyd, 2005). To produce biomass for DNA sequencing, ~50 FeS gradient plates (15–18 ml each) were harvested for each strain. The cells and iron oxides were harvested by centrifugation and the cell/iron oxide pellet was washed once with phosphate buffer (50 mM, pH 8.0) and DNA was extracted using the MoBio PowerSoil DNA kit to a quantity (~70 μg) and quality specified by the JGI.

### Genome sequencing, annotation, and analysis

Genome sequencing was done at the JGI using a combination of Illumina (Bennett, 2004) and 454 technologies (Margulies et al., 2005). An Illumina GAii shotgun library with reads of 121 Mb (ES-1) and 244 Mb (ES-2), a 454 Titanium draft library with average read length of 195 bp (ES-1) and 248 bp (ES-2) and a paired end 454 library with average insert size of 12.4 Kb (ES-1) and 11 Kb (ES-2) were generated. Illumina sequencing data was assembled with VELVET (Zerbino and Birney, 2008), and the consensus sequences were shredded into 1.5 kb overlapped fake reads and assembled together with the 454 data. The draft assembly for ES-1 was based on 129.0 Mb of 454 draft data, and 114 kb of 454 paired end data. The ES-2 draft was based on 117.1 Mb 454 draft data, and all of the 454 paired end data. The initial Newbler assembly contained 12 contigs in 1 scaffold for ES-1, and 54 contigs in 2 scaffolds for ES-2. This 454 assembly was converted into a phrap assembly by making fake reads from the consensus, collecting the read pairs in the 454 paired end library. The Phred/Phrap/Consed software package (www.phrap.com) was used for sequence assembly and quality assessment (Ewing and Green, 1998; Ewing et al., 1998; Gordon et al., 1998) in the following finishing process. Illumina data was used to correct potential base errors and increase consensus quality using a software Polisher developed at JGI (Alla Lapidus, unpublished). After the shotgun stage, reads were assembled with parallel phrap (High Performance Software, LLC). Possible mis-assemblies were corrected with gapResolution (Cliff Han, unpublished), Dupfinisher (Han and Chain, 2006), or sequencing cloned bridging PCR fragments with subcloning. Gaps between contigs were closed by editing in Consed, by PCR and by Bubble PCR primer walks. A total of 96 (ES-1) and 297 (ES-2) additional PCR reactions were necessary to close gaps and to raise the quality of the finished sequence for both genomes.

Genes were identified using Prodigal (Hyatt et al., 2010). The predicted CDSs were translated and used to search the National Center for Biotechnology Information (NCBI) non-redundant database (nr), UniProt, TIGRFam, Pfam, KEGG, COG, and InterPro databases. The tRNAScan-SE tool (Hacker and Kaper, 2000) was used to find tRNA genes, whereas ribosomal RNA genes were found by searches against models of the ribosomal RNA genes built from SILVA (Pruesse et al., 2007). Other non–coding RNAs such as the RNA components of the protein secretion complex and the RNase P were identified by searching genomes for the corresponding Rfam profiles using INFERNAL (Makarova et al., 1999). Additional gene prediction analysis and manual functional annotation was performed within the Integrated Microbial Genomes (IMG) (Markowitz et al., 2008) platform developed by the JGI (http://img.jgi.doe.gov).

Primary genome analysis was done using the JGI's IMG/ER web-based set of tools for gene comparison and annotation. For the phylogenetic analyses presented, protein sequences were obtained from the NCBI database and aligned with ClustalW using the BLOSUM matrix and further aligned by hand. Maximum likelihood phylogenetic trees were created in MEGA5 (Tamura et al., 2011) using the Poisson Substitution Model and 1000 bootstrap iterations.

### Growth studies

Alternative growth substrates to Fe(II) were either tested in gradient tubes, or by inoculating the cells into sealed serum bottles (50 ml in a 125 cc serum bottle) containing liquid MWMM medium amended with the particular growth substrate (Emerson and Floyd, 2005). The headspace contained 2–3% oxygen in a N_2_-atmosphere, and the pH was buffered to 6.5 with 10 mM MES buffer. For nitrate dependent growth, the MWMM medium was supplemented with 10 mM NaNO_3_, and the headspace was maintained O_2_ free. To assess growth on thiosulfate, the MWMM medium in serum bottles was amended with 5 mM thiosulfate and 2–3% O_2_ was maintained in the headspace. In all cases, growth was assessed by light and epifluorescence microscopy, either qualitatively or quantitatively by direct cell counts as described previously (Emerson and Moyer, 1997).

### Fatty acids analysis

Fatty acid methyl esters (FAME) were identified using the procedure recommended by the Microbial Identification System (MIDI, Sherlock Microbial Identification System Version 4.0, MIS Operating Manual, March 2001, Newark, DE). The cells were grown in FeS gradient plates until the late log phase of growth and then harvested by centrifugation. Excess Fe(III) was removed from the cell pellet by treating the sample with 0.33 M oxalic acid for 1 h at 37°C, and then washing it three times by centrifugation with de-ionized water. The extraction of FAMEs and their analysis by gas chromatography was done as previously described (Pikuta et al., 2003).

## Results and discussion

### Genome properties and phylogeny

**Figure 1:**
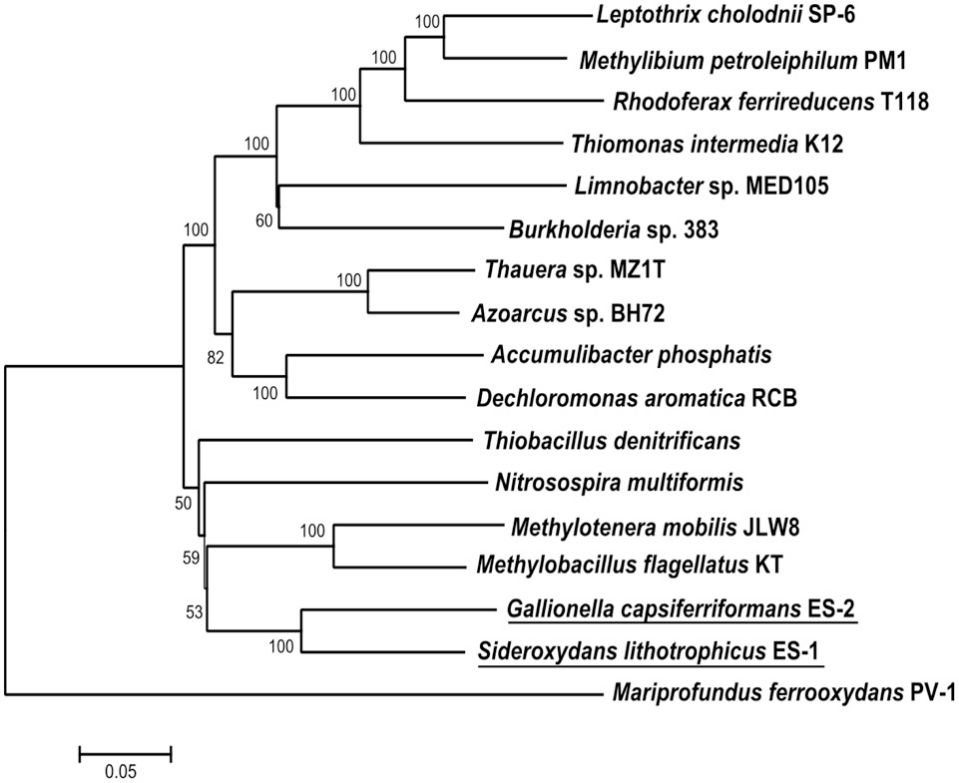
**Maximum likelihood phylogenetic tree of 6 concatenated conserved proteins (RpoA, RpoB, RecA, GyrB, FusA, and IleS) within *Sideroxydans lithotrophicus* ES-1 and *Gallionella capsiferriformans* ES-2 as well as other Betaproteobacteria**. *M. ferrooxydans* PV-1, a member of the Zetaproteobacteria was used as the outgroup.

Both ES-1 and ES-2 have single circular chromosomes of ~3 Mb as shown in Table 1. The genome sizes are consistent with those of other aerobic, chemolithoautotrophic FeOB, *Acidithiobacillus ferrooxidans* (2.9–3.0 Mb), *Mariprofundus ferrooxydans* (3.0 Mb), and *Leptospirillum ferrooxidans* C2–3 (2.56 Mb; Valdés et al., 2008; Singer et al., 2011; Fujimura et al., 2012). Neither genome shows evidence for extrachromosomal elements; nor is there evidence for large blocks of genes (e.g., “pathogenicity islands”) that may have been introduced by horizontal gene transfer (HGT; Gogarten and Townsend, 2005). ES-2 has three SSU rRNA genes that share 100% identity to one another, while ES-1 has two SSU genes, also sharing 100% identity. *Gallionella* ES-2 and *Sideroxydans* ES-1 are relatively distant from one another based on comparison of their SSU rRNA genes, sharing 93% similarity, well below the current standard of 98.7% for differentiating species (Stackebrandt and Ebers, 2006). They also differ in G+C content, Table 1. There is limited synteny between the two chromosomes. A phylogeny based on the SSU rRNA gene indicated these two FeOB cluster with numerous environmental clones from freshwater Fe-rich habitats, other isolates of FeOB (Weiss et al., 2007; Ludecke et al., 2010; Krepski et al., 2012), and form a distinct clade within the Betaproteobacteria (data not shown). A more robust multi-locus sequence analysis (MLSA) used six phylogenetically conserved genes (*rpoA, rpoB, recA, gyrB, fusA*, and *ileS*) to compare the 10 most closely related members of the Betaproteobacteria that have complete genome sequences (Figure 1). This confirmed the position of these two FeOB as being most closely related to one another and forming a distinct clade in the Betaproteobacteria that could form a novel order, the Gallionellales. Overall the two genomes share significant homology, at a 30% identity ~60% of the genes are homologous (Table 1), and at 60% identity, about 40% of the genes are homologous. This genomics based approach also confirms the phylogenetic distance to the marine Fe-oxidizer *M. ferrooxydans*, which belongs to the Zetaproteobacteria, a proposed novel class of Proteobacteria (Emerson et al., 2007). Despite this lack of phylogenetic relatedness, the microaerophile *M. ferrooxydans* shares common physiological traits with ES-1 and ES-2, and common morphological traits with ES-2.

**Table 1:** **Genome characteristics of ES-1 and ES-2**.

**Organism**	***Sideroxydans* ES-1**	***Gallionella* ES-2**
Genome size	3.00 Mb	3.16 Mb
Genes	3049	2977
CDS	2996 (98.26%)	2917 (97.98%)
GC Perc	58%	53%
16S genes	2	3
tRNA genes	44	51
Genes w/functional prediction	2177 (71.4%)	2297 (77.16%)
COGs	2237 (73.4%)	2157 (72.5%)
Pseudo genes	16	0
CRISPR-related genes	0	0
% homologous genes (1e-5; 30%)	63.7%	64.5%

### Energy transduction from iron

As previously reported, both strains grow on Fe(II) with optimal doubling times in FeS gradient tubes of 8 h (ES-1) to 12 h (ES-2; Emerson and Moyer, 1997). Follow up studies reported here indicated the source of Fe(II) used for growth mattered little. In gradient culture, both strains grew on FeCO_3_ (siderite), and in liquid culture they grew with FeCl_2_, Fe(NH_4_)_2_(SO_4_)_2_, and FeSO_4_, all with comparable results. Neither strain was able to couple nitrate reduction to iron oxidation, nor were they able to reduce Fe(III) in the presence of acetate under anaerobic conditions. Neither strain could grow on complex media, e.g., R2A, nutrient broth, or peptone, yeast extract, glucose (PYG, ATCC medium #1503), or utilize acetate, pyruvate, succinate, glucose, galactose, ribose, glycerol, aspartate, or glycine as a sole growth substrate in a minimal medium.

#### Potential candidates for an iron oxidase

In order to capitalize on Fe(II) as an energy source, an organism needs to couple electrons gained through the oxidation of ferrous to ferric iron to the electron transport chain to generate proton motive force (pmf). It is assumed that this initial oxidation is carried out by an iron-oxidizing protein at the cell surface, presumably located at the cell outer membrane, since, at circumneutral pH, Fe(III) reacts almost instantaneously with water to produce insoluble ferric oxyhydroxide (Stumm and Morgan, 1981). Thus, the cell requires a mechanism for extracellular electron transfer from the site of Fe-oxidation to the electron transport chain on the cytoplasmic membrane.

**Figure 2:**
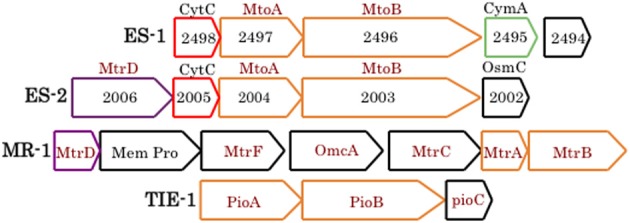
**Gene clusters from ES-1, ES-2, *S. oneidensis* MR-1, and *R. palustris* TIE-1 that are involved in Fe-oxidation or Fe-reduction (the Mtr cluster in MR-1)**. The *mtoA/B* genes are homologs of *mtrA/B* and *pioA/B*, all outlined in orange. ES-1 has a *cymA* homolog (4e-21), green outline, to the *cymA* gene in MR-1 (not shown), and ES-2 and MR-1 share a weakly homologous *mtrD*, outlined in purple. Homologous genes between ES-1 and ES-2 that encode a cytochrome C are outlined in red. Genes outlined in black are non-homologous. The numbers refer to the respective Slit_xxxx or Galf_xxxx locus tags in IMG. The respective IMG locus tags for respective genes in MR-1 are SO1782 – SO1776, and for TIE-1, Rpal_0817 – Rpal_0815.

At present, specific mechanisms about how cells conserve energy from oxidation of Fe(II) at circumneutral pH are poorly understood (Emerson, 2012; Ilbert and Bonnefoy, 2013). Recently it has been pointed out that strains ES-1 and ES-2 contain gene homologs to the genes encoding MtrA/B in *Shewanella oneidensis* MR-1, and the PioA/B genes in *Rhodopseudomonas palustris* TIE-1 that are involved in Fe-reduction and photoferrotrophy, respectively (Liu et al., 2012). Experimental evidence has shown that the MtrA homolog in ES-1, designated MtoA (metal oxidation), produces a decaheme cytochrome with Fe(II) oxidation activity *in vitro*. In concert with MtoB (the MtrA homolog) and a CymA homolog found in the same operon, it was proposed these genes could form an Fe-oxidizing pathway in strain ES-1 (Liu et al., 2012). In *S. oneidensis*, CymA is a tetraheme c-type cytochrome that is thought to reside in the cytoplasmic membrane and mediate electron transfer from the quinone/quinol pool (Gralnick, 2012; Coursolle and Gralnick, 2012; Shi et al., 2012). The relevant gene clusters are shown in Figure 2. ES-2 has MtoA/B (Galf_2004 and Galf_2003) homologs in a gene cluster that shares a c-type cytochrome upstream of MtoA/B with ES-1, but also has an additional multiheme cytochrome, immediately upstream of the c-type cytochrome. This gene (Galf_2006) does not have a homolog in ES-1, but has weak homology (bit score 47; E value 6e-06) to MtrD, a decaheme cytochrome in *S. oneidensis* MR-1 (Coursolle and Gralnick, 2012). *Gallionella* strain ES-2 does not have a CymA homolog. These genomic patterns suggest that while ES-1 and ES-2 share the MtoA/B genes, the specific functioning of these genes may be different in the two organisms.

The most well-characterized pathway for aerobic Fe-oxidation is in the acidophile *A. ferrooxydans* (ATCC 23270); the gene *cyc2* encodes a cytochrome located in outer membrane of the cell that catalyzes oxidation of Fe(II) to Fe(III); (Bonnefoy and Holmes, 2011). Electrons released during this oxidation can flow “downhill” to a terminal cytochrome oxidase via a cytochrome encoded by the *cyc1* gene, or flow “uphill” to the quinone pool and an the NADH synthase complex. This is, in part, mediated by another cytochrome encoded by the *cycA1* gene. It is also thought rusticyanin plays a key role in electron shuttling from the outer membrane to the cytoplasmic membrane, and may also control “uphill” vs. “downhill” flow of electrons (Bird et al., 2011; Bonnefoy and Holmes, 2011). Neither *Sideroxydans* ES-1 or *Gallionella* ES-2 contain homologs to rusticyanin, Cyc1, or CycA1. *Sideroxydans* ES-1 has three weak homologs (E values 8 × 10^−9^ to 1 × 10^−4^) to the *cyc2* gene that occur in a cluster of three genes (Slit_0263 – Slit_0265); however these genes are not associated with other redox active genes and do not have transmembrane helices. There is one copy of a *cyc2* gene homolog in ES-2 (Galf_0431) that shares homology to the genes in ES-1.

A previous report on the genome of *M. ferrooxydans* PV-1, noted that all three strains of FeOB, PV-1, ES-1, and ES-2 contained molybdopterin oxidoreductase (*actB*)-containing gene cluster recognized as the alternative complex (AC) III (Pereira et al., 2007; Singer et al., 2011). This was the only homologous multiple gene cluster shared between these three organisms that could play a role in redox reactions directly coupled to Fe-oxidation. PV-1 does not possess Mto homologs. Recent work of Refojo et al. (2012) indicates the *actB* gene, which was proposed to act as a putative Fe oxidase (Singer et al., 2011), is likely bound to the cytoplasmic membrane in *Rhodothermus marinus*. If this is the case in the Fe-oxidizers then it is less clear how it would act as an Fe-oxidase, although it may still be involved in electron transport (see below).

**Figure 3:**
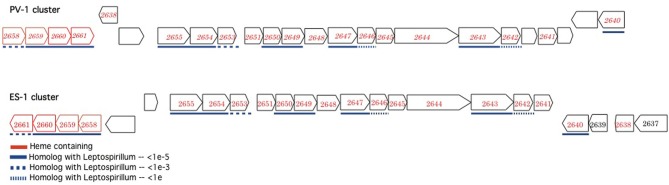
**Complementary gene clusters of potentially redox active proteins shared between *Sideroxydans* ES-1 and *M. ferrooxydans* PV-1, some of which are also homologous to *Leptospirillum* spp**. The numbers designate the IMG gene locus for the respective genes in *Sideroxydans* (Slit_xxxx), in the PV-1 gene clusters the homologous ES-1 gene is shown in italics. The genes outlined in red contain hemes.

In addition to the ACIII complex, *Sideroxydans* ES-1 and *M. ferrooxydans* share a suite of 20 genes in common that are organized into three nearly contiguous clusters, with highly conserved gene order (Figure 3). The closest match by BLAST for each of the ES-1 genes is the cognate PV-1 gene (results not shown). Remarkably, 14 of these genes also share homology with the acidophilic Fe-oxidizer *Leptospirillum ferrooxidans*, in fact in every case, for these homologous genes the next closest match after PV-1 is to a gene from a *Leptospirillum* spp. The cognate genes in *L. ferrooxidans* C2–3 do not share the same highly conserved gene order as is found in ES-1 and PV-1 (Fujimura et al., 2012). The majority of these genes are annotated as hypotheticals, although some of them have redox properties, thus their function remains unknown. *Gallionella* ES-2 does not possess these genes.

#### Electron transport complexes

**Figure 4:**
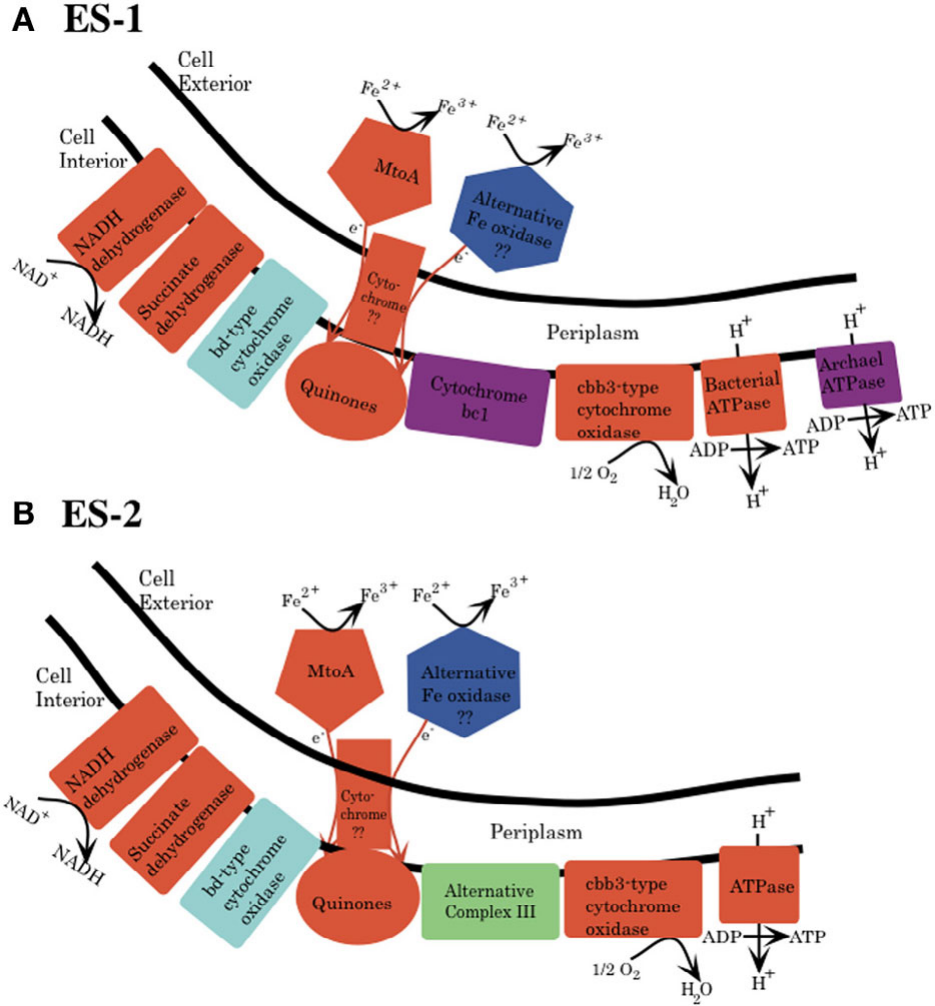
**Schematic for electron transport from Fe(II) to the electron transport chain in the cytoplasmic membrane for both *Sideroxydans* ES-1 (A) and *Gallionella* ES-2 (B)**. This is based solely on genomic analysis. MtoA is shown as one possible protein that initiates oxidation of Fe(II) to Fe(III), and an alternative oxidase is also shown, but there is no specific candidate for this protein yet. The mechanism for transfer of electrons from the outer membrane to the cytoplasmic membrane is also unknown. **(A)** Cytochrome bc1 (purple) and archaeal type ATPase are found only in ES-1. **(B)** ES-2 lacks cytochrome bc1, and it is proposed that the ACIII could substitute this function. Both organisms have a bd-type cytochrome oxidase, which could interact directly with the quinones. The specific stoichiometry of proton flux across the cytoplasmic membrane is not understood and is not shown.

The electron transport chain responsible for generation of pmf and ATP generation consists of five separate complexes (Nicholls and Ferguson, 2002). Both ES-1 and ES-2 have complete gene complements for the NADH dehydrogenase (synthase; Complex I), as well as complex II, succinate dehydrogenase, Figure 4. Both organisms have quinones that couple electron transport from the NADH dehydrogenase to complex III type cytochromes; however here the pathways between ES-1 and ES-2 show divergence. ES-1 has genes (Slit_130 – Slit_132) for a canonical cytochrome *bc*_1_ complex; however ES-2 does not contain such homologs. As discussed above, both organisms do contain an eight gene cluster that includes a molybdopterin oxidoreductase. This ACIII complex is capable of interacting with the quinone pool and passing electrons to a terminal oxidase (Refojo et al., 2012). If this is the case, then ES-2 may utilize this in place of the *bc*_1_ complex. Both ES-1 and ES-2 also encode a *cbb*_3_ type terminal cytochrome oxidase (C-family heme-copper oxidoreductases), but lack the cytochrome *aa*_3_ or *bo*_3_ terminal oxidases (A-family) that have low affinity for O_2_ (Gennis and Stewart, 1996). This is consistent with these organisms being obligate microaerophiles, since the *cbb*_3_ cytochrome oxidase has a very high affinity for oxygen, while bo type oxidases are thought to be most efficient in more oxic conditions (Han et al., 2011; Morris and Schmidt, 2013). Both ES-1 and ES-2 also encode cytochrome bd oxidases, which also have a high oxygen affinity, and could serve as terminal oxidases coupled directly to the quinone pool (Borisov et al., 2011). Finally, ES-1 and ES-2 both encode genes for a canonical bacterial ATP synthase coded in contiguous 8 gene clusters (Slit_2980 – Slit_2987 and Galf_2932 – Galf_2939, respectively). ES-1, alone, has a second bacteria/archaea ATP synthase. The subunits for this ATPase are in a contiguous gene cluster (Slit_2558 – Slit_2550). The genes for this latter ATPase share highest homology with an ammonia-oxidizing *Nitrococcus* spp. The advantage of having this second ATP synthase is not known.

### Sulfur oxidation

**Figure 5:**
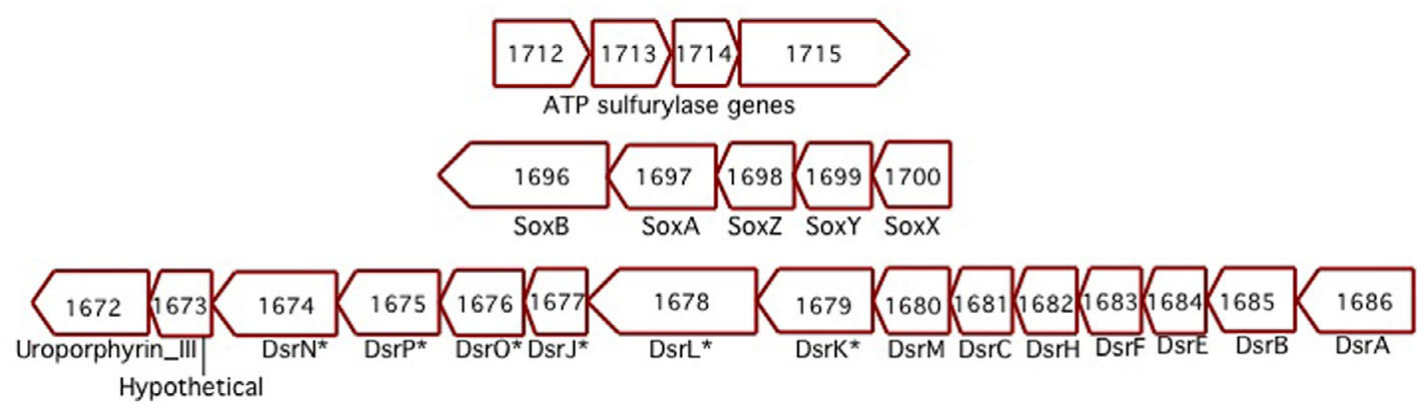
**Three separate clusters of sulfur-oxidizing genes found in ES-1**. The Dsr-homologs that are denoted with an ^*^ are respective gene designations that are listed only in *Allochromatium*. The numbers designate the IMG gene locus for ES-1, Slit_xxxx.

*Sideroxydans* ES-1 has a *soxXYZAB* gene cluster (Slit_1700 – Slit_1696), these genes share significant homology and gene order to the *soxXYZAB* genes in *Thiobacillus denitrificans* (Figure 5), and are generally thought to be involved in growth on sulfide or thiosulfate (Ghosh and Dam, 2009). In the same genomic region are 15 contiguous genes (Slit_1671 – Slit_1686) that encode for the alpha and beta-subunits of dissimilatory sulfite reductase, the *dsrEFHC* genes, and other genes that appear to be involved in lithotrophic S-metabolism (Ghosh and Dam, 2009). These genes share the same order and are highly homologous to a gene cluster in *T. denitrificans*, as well as sharing the same gene order with *Allochromatium vinosum* (DSM 180). The presence of these suites of S-oxidation genes suggest ES-1 should be able to grow by utilizing reduced S-compounds.

**Figure 6:**
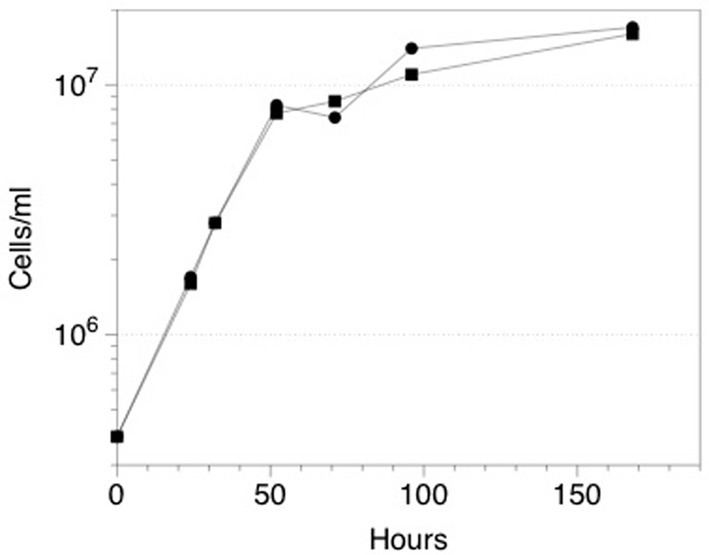
**Growth of *Sideroxydans* ES-1 on 5 mM thiosulfate (•), or FeCl_2_ (▪)**. Each time point is the result of a duplicate sample, the standard error was 10–15% for each of the values; error bars are not shown to maintain clarity.

Although initial attempts to grow ES-1 on thiosulfate or sulfide were unsuccessful (Emerson and Moyer, 1997), a subsequent attempt to grow ES-1 on 5 mM thiosulfate in a mineral salts medium was successful (Figure 6). The doubling time (approximately 8 h) of cells growing on thiosulfate was about the same as cells grown on FeCl_2_, which is surprising since the free energy available from thiosulfate is several times greater than for Fe(II); (White et al., 2012). This implies *Sideroxydans* ES-1 is better adapted for growth on Fe(II) than reduced S-compounds; however the ability to grow on these compounds would give it a competitive advantage in ecosystems where reduced S-compounds were also present. *Gallionella* ES-2 does not possess S-oxidation pathways, nor does it show evidence for growth on reduced S compounds. A search of the literature and analysis of Genbank records did not reveal any evidence for *Sideroxydans* or Gallionellales environmental 16S sequences from S-rich freshwater habitats, e.g., caves or freshwater sulfureta, again suggesting S-oxidation is not a preferred metabolism for this group of organisms.

### CO_2_ fixation

**Figure 7:**
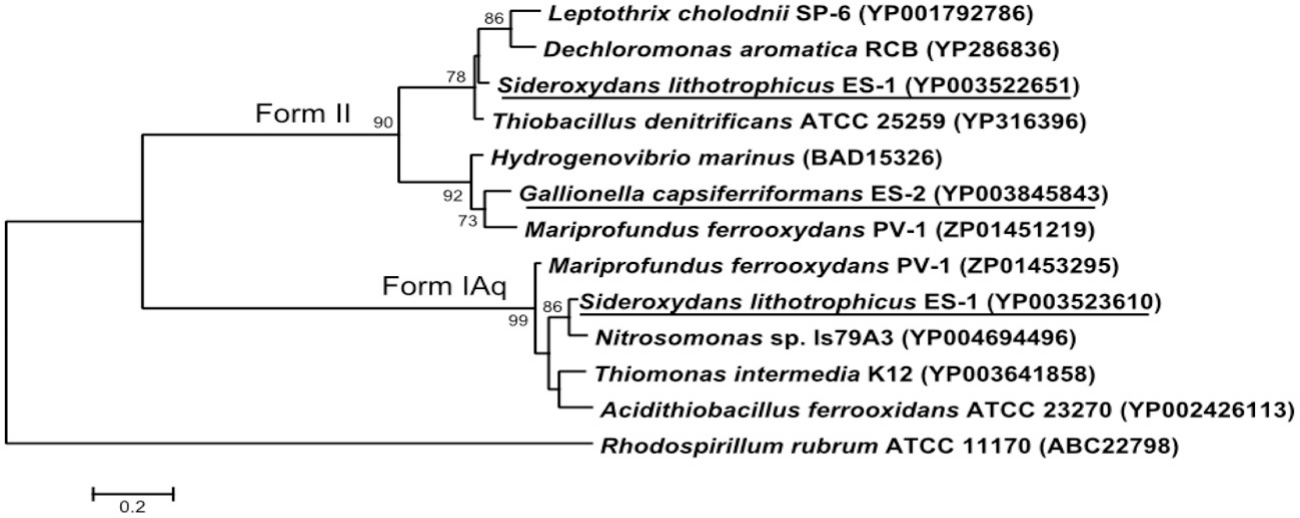
**Maximum likelihood phylogenetic tree of the Form II Ribulose bisphosphate carboxylase (RubisCO) large subunit proteins within *S. lithotrophicus* ES-1 (Slit_0022), and *G. capsiferriformans* ES-2 (Galf_0034), and the small subunit Form IAq gene from ES-1 (Slit_0968)**. Related Betaproteobacteria are also shown, but both RubisCO genes also share a closer than expected relationship with *M. ferrooxydans* PV-1. The Alphaproteobacteria *Rhodospirillum rubrum* represents the out-group and only bootstrap values above 50% are reported.

Both strains ES-1 and ES-2 contain ribulose 1,5-bisphosphate carboxylase (RubisCO) genes. Strain ES-1 has two large subunit RubisCO genes. One of these genes (Slit_0022) is in a 7 gene cluster with cbbQ, cbbO, fructose-1-6-bisphosphatase, and a carbonate dehydratase (likely a carbonic anhydrase). This 7 gene cluster is identical in gene order and composition to one in strain ES-2 (Galf_0034 – Galf_0040). This is the only RubisCO gene cluster in ES-2. While these Form II RubisCO proteins are homologous to other close relatives within the Betaproteobacteria, such as *Thiobacillus denitrificans*, they also share significant similarities to non-related microorganisms such as *Hydrogenovibrio marinus* and *Mariprofundus ferrooxydans* PV-1 (Figure 7).

*Sideroxydans* ES-1 possesses a second large subunit RubisCO (Slit_0985) in a 5 gene cluster (Slit_0985 – Slit_0989) that includes a small subunit RubisCO (Slit_0986), cbbQ, cbbO, and a small (100aa) hypothetical protein. The large subunit of this RubisCO operon is most homologous with other chemolithoautotrophs including *Nitrosomonas* spp., *Acidithiobacillus ferrooxidans*, and *M. ferrooxydans* PV-1. The small subunit RubisCO is in the Form IAq RubisCO group (Badger and Bek, 2007), and is homologous to *Thiobacillus* spp., *Nitrosomonas* spp. and *M. ferroxydans* (Figure 7). This type of RubisCO is not known to be associated with carboxysomes, consistent with there being no evidence for carboxysome genes in ES-1, nor has transmission electron microscopy revealed any obvious carboxysome-like structures in these cells (Emerson and Moyer, 1997).

The Form I enzymes have a higher affinity for CO_2_ compared to O_2_, which would allow them to fix CO_2_ at higher O_2_ partial pressures than organisms with Form II only (Badger and Bek, 2007). In strictly microaerophilic microorganisms, Form II RubisCO may rarely see oxygen levels high enough to stimulate the oxygenase reaction compared to the CO_2_ fixation pathway. The fact that strain ES-1 has both Form I and Form II RubisCO, suggests it is more tolerant of fluctuating, and overall higher O_2_ levels, than strain ES-2.

### Hemerythrins and globins

Hemerythrin and globin genes are found in many bacteria, and can be involved in oxygen-sensing and oxygen-binding. They may be also be involved in oxygen storage, oxygen-sensing, detoxification, or even the binding of iron or other metals (French et al., 2008). Both ES-1 and ES-2 have single-domain and multi-domain hemerythrin genes; however strain ES-1 has significantly more total hemerythrin genes than strain ES-2 (14 and 3, respectively; Table 2). This difference is attributed to the 11 single-domain hemerythrins that ES-1 possesses (ES-2 has only one). More specifically, ES-1 has 10 metal-binding hemerythrins, one type of single-domain hemerythrin, while ES-2 has none. Strain ES-1 also has significantly more total hemerythrin genes when compared to other circumneutral pH FeOB such as *Leptothrix cholodnii* SP-6 and *Mariprofundus ferrooxydans* PV-1 (5 and 2, respectively), Table 2. The only other organisms known to have as many metal-binding single-domain hemerythrins as ES-1 are magnetotactic bacteria such as *Magnetospirillum magneticum*, where these genes are believed to be important in aerotaxis (Bailly et al., 2008; French et al., 2008). Hemerythrins involved in the binding of oxygen typically have 6 specific hydrophobic amino acid residues that line the oxygen-binding pocket (French et al., 2008). Of the 10 metal-binding hemerythrins found in strain ES-1, 8 of them have all 6 of these conserved hydrophobic residues, suggesting they bind to O_2_. The HHE cation-binding single-domain hemerythrin genes (another type of single-domain hemerythrin) found in ES-1 and ES-2 does not have these conserved hydrophobic residues suggesting they may not bind O_2_, but could instead bind iron, or another metal. ES-1 and ES-2 also contain three and two multi-domain hemerythrins, respectively. These also contain conserved hydrophobic residues. Many of the multi-domain hemerythrins are found in gene clusters with large numbers of chemotactic and environmental sensing genes (see Table 2) suggesting they may likely be involved in oxygen-sensing in the environment.

**Table 2:** **Summary of hemerythrin-like protein types in circumneutral iron-oxidizing bacteria**.

**Organism**	**Single-domain**		**Multi-domain**				**Total hemerythrin-like genes**
	**Metal-binding**	**HHE cation-binding**	**PAS/PAC**	**GGDEF**	**Histidine Kinase**	**Methyl-accepting chemotaxis**	
*Sideroxydans lithotrophicus* ES-1	10	1	2	0	1	0	14
*Gallionella capsiferriformans* ES-2	0	1	1	0	1	0	3
*Mariprofundus ferrooxydans* PV-1	0	0	0	1	0	1	2
*Leptothrix cholodnii* SP6	1	4	0	0	0	0	5

**Figure 8:**
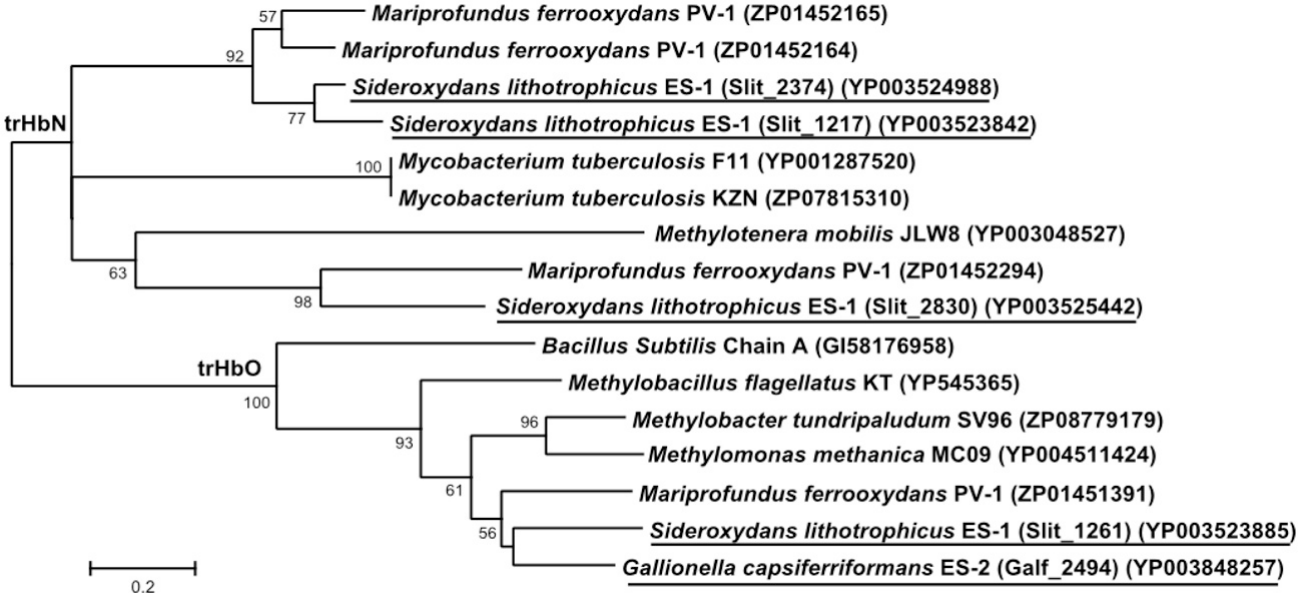
**Maximum likelihood phylogenetic tree of truncated hemoglobin (trHb) proteins showing the close relationship of *Sideroxydans lithotrophicus* ES-1, *Gallionella capsiferriformans* ES-2, to *Mariprofundus ferrooxydans* PV-1, and compared to reference organisms, and other related Betaproteobacteria**. Only bootstrap values above 50% are reported.

Both strains ES-1 and ES-2 also contain genes for truncated hemoglobins (trHb), a short length hemoglobin often found in bacteria, which binds to O_2_. Strain ES-1 possesses a total of 4 trHb genes which is greater than the single globin gene found in most organisms with similar genome sizes (Vinogradov et al., 2006). The average genome size for organisms with four globin genes is 6.5 ± 1.9 Mb, more than double the genome size of strain ES-1 suggesting these globins are important for strain ES-1's survival. The four trHbs found in ES-1 represent two major types: trHbO (Slit_1261) and trHbN (Slit_1217, Slit_2374, Slit_2830). Strain ES-2 contains only one trHbO gene (Galf_2494). Due to differences in O_2_ binding efficiencies, it has been proposed that trHbO proteins are involved in O_2_ transport under microaerophilic conditions in other organisms while trHbN proteins may be part of a terminal oxidase or involved in nitric oxide detoxification (Pathania et al., 2002; Lama et al., 2006; Niemann and Tisa, 2008). Both strain ES-1 and ES-2 may use trHbO for improving O_2_ transfer efficiency of aerobic respiration under microaerophilic conditions. Interestingly, the trHbN proteins of strain ES-1 are more highly similar to those of *M. ferrooxydans* PV-1 (which also has three copies of trHbN and one copy of trHbO) than to other related Betaproteobacteria, suggesting either a common ancestor, or a strong common selective pressure for the genes from ES-1 and PV-1 (Figure 8). Both ES-1 and ES-2 have nitric oxide reductases suggesting the trHbN found in strain ES-1 is more likely a part of a terminal oxidase rather than involved in NO detoxification. Similarly, the trHbO protein from ES-1, ES-2, and PV-1 are all most similar to each other (~60% amino acid similarity), further demonstrating the potential importance of these oxygen-binding proteins for FeOB that live under microaerophilic conditions with high concentrations of Fe(II).

Why strain ES-1 has so many hemerythrin and globin genes remains unknown. It is quite likely that these genes are involved in oxygen-sensing in the environment as has been suggested for magnetotactic bacteria (Bailly et al., 2008; French et al., 2008). Another intriguing hypothesis is that ES-1 could use these proteins to store oxygen and then grow by Fe-oxidation under anoxic conditions. This would allow it to access higher Fe(II) concentrations in anoxic water, or continue to oxidize Fe(II) under conditions where O_2_ concentrations fluctuated between oxic and anoxic. Alternatively, these genes may play a part in the overall strategy that ES-1 utilizes to protect itself from significant fluctuations in environmental oxygen concentrations, by either binding excess O_2_ in the cytoplasm or Fe(II) and limiting the production of reactive oxygen species. If it is more commonly exposed to these types of fluctuations or is just more sensitive to them is unclear; however, in either case this could explain why ES-1 has so many more of these genes compared to ES-2.

### Motility, chemotaxis, and environmental sensing mechanisms

Both *Sideroxydans* ES-1 and *Gallionella* ES-2 have significant percentages of genes involved in environmental sensing and signal transduction. ES-1 has 9.3% of its genes in the COG category, signal transduction mechanisms, while ES-2 has 11.6% of its COGs in this category. These numbers are higher than for other Betaproteobacteria like the methylotrophs, *Methylotenera* spp. (8%), and *Methylobacillus flagellatus* (6.4%), or ammonia-oxidizing *Nitrosomonas* spp. (5%) They are also substantially higher than the 7% average for signal transduction genes estimated for marine copiotrophs, and nearly triple the 3.6% average for oligotrophic marine microbes as reported by Lauro et al. (2009). These COG percentages are similar to the Fe-reducing *Geobacter* spp. (around 10%), and a little lower than some magnetotactic bacteria (around 12.5%) that live in redox stratified environments.

The 10 most abundant COGs in ES-1 and ES-2 are listed in Table 3. In ES-1 seven of these COGs are involved in signal transduction, chemotaxis, or some type of environmental sensory response, while in ES-2, eight of the top 10 COGs are related to these activities. They share six of these top 10 COGs in common, all related to sensory genes. COGs 5002 and 2199 that encode for GGDEF and EAL domains in sensory proteins account for 43 genes in ES-1, and 46 genes in ES-2. Of the 2366 genomes listed in IMG at the time of this study that contain COG 2199, only 42 genomes have a greater number of these genes than ES-2; 19 of these are from *Vibrio* spp. that all have significantly larger genomes than ES-2. Other genomes with high numbers of COG2199 include sulfur-oxidizing bacteria in the Epsilonproteobacteria, including Campylobacterales sp. GD1 (54 genes), and *Sulfuricurvum kujiense* YK-1 (38 genes). These chemolithoautotrophic S-oxidizers also thrive in redoxicline habitats. GGDEF domains control synthesis of the secondary messenger cyclic di-GMP (C-di-GMP) by diguanylate cyclase, while the EAL domain is responsible for degradation of C-di-GMP through phosphodiesterase activity (Römling, 2011). Through their actions with C-di-GMP these domains are involved in eliciting physiological responses to a variety of external signals including oxygen, and nutrient starvation, as well as controlling cellular behavior and differentiation between sessile, e.g., biofilm formation, and motile states (Jenal and Malone, 2006). The majority of work on these signaling systems has been done on a few model organisms, mostly in relation to virulence, and it is likely they respond to many more environmental signals that have yet to be identified (Jenal and Malone, 2006).

**Table 3:**
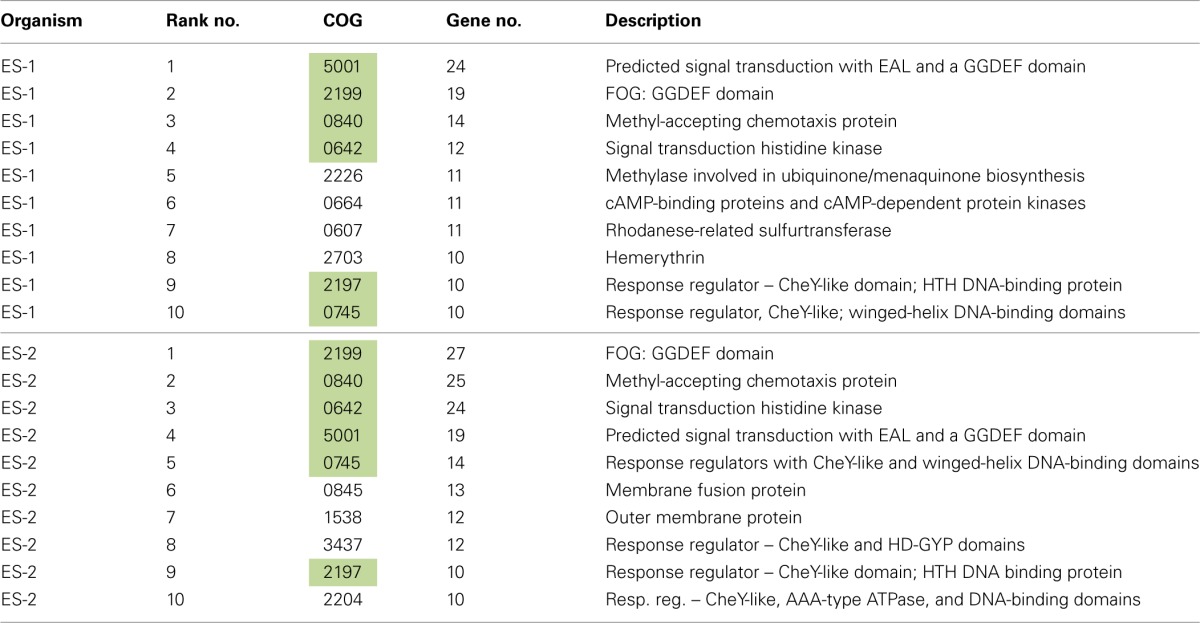
**Ten most abundant COGs in ES-1 and ES-2**.

*Shaded COGs are present in both organisms*.

Histidine kinases (HK) are another class of enzymes that are known to play an important role in environmental signaling and gene regulation (White et al., 2012). ES-1 has 37 HK genes, and ES-2 has 47. These numbers place them in the top 10% for HK gene abundance among currently sequenced bacterial genomes. While there is overlap in their HK genes, there are also differences in the types of HKs the two organisms possess. ES-1 has 17 (46%) of its HK genes associated with two component regulatory systems, including 7 luxR family proteins, as well as three CheA HK's. ES-2 has five CheA HK's, but only 30% (14 genes) of its HK's are associated with two component regulatory systems, with three of these belonging to the LuxR family. For ES-2, 38% of its HK's are associated with other types of sensor elements that include unidentified regulatory response receivers, PAS/PAC type sensors, and others. Thus, it appears that while HK's must play an important role in extracellular sensing and gene regulation for both organisms, different signaling mechanisms and types of response may be elicited by the HK's.

The most well-understood behavioral trait among bacteria is the ability to produce flagella, and control motility to track gradients of chemo-attractants or chemo-repellents (Hazelbauer et al., 2008). In growing cultures of *Sideroxydans* ES-1 and *Gallionella* ES-2 motility is a variable trait, at certain times motile cells are observed; but are not always present, and as yet, motility has not been shown to correspond with any particular phase of growth for either organism. Consistent with the observation of motile cells, both strains contain nearly complete sets of flagellar genes. In ES-2 these are encoded in contiguous gene clusters (Galf_1042 – Galf_1079), while in ES-1 there are two separate clusters (Slit_0562 – Slit_0575 and Slit_0568 – Slit_0599) in close proximity to one another.

Both ES-1 and ES-2 will grow as distinct bands in opposing gradients of oxygen and Fe(II), and appear to be able to track these gradients (Druschel et al., 2008), suggesting they are tactic to O_2_ and/or Fe(II). Specific chemotaxis assays have not been done for either strain, however both organisms contain full complements of chemotaxis genes. ES-2 has redundant copies of most chemotaxis genes, including five *cheA* histidine kinase genes; six *cheW* genes, four *cheZ* genes, and 2 *cheY* genes that control flagellar switching (Porter et al., 2011). ES-1 has fewer total chemotaxis genes, but still has redundant copies of *cheA*(2), and *cheY*(2). ES-2 has 25 methyl-accepting chemotaxis genes (MCPs), while ES-1 has 14 MCPs. The MCPs are known to act as chemoreceptors that bind specific attractants or repellents and initiate a tactic response through coordination with Che genes (Hazelbauer et al., 2008). These are relatively large numbers of MCPs, for example, *Escherichia coli* has 5 chemoreceptors while *Pseudomonas aeruginosa* has 26 receptors, but its genome is twice the size of ES-2 (Porter et al., 2011). In terms of responding tactically to oxygen (Taylor et al., 1999), ES-2 has three aerotaxis (aer) genes (Galf_0774, Galf_1969, and Galf_2093), while ES-1 has one aer gene (Slit_0546).

Overall, both organisms have a rich complement of genes for environmental sensing, motility, and chemotaxis, indicating that they are capable of an array of behavioral and physiological responses for adapting to a dynamic environment. The signaling pathways implied by these families of genes are complex, and based on studies of model organisms could include chemotaxis-like pathways that are involved in processes like biofilm formation or other behaviors (Porter et al., 2011). Members of the Fe-reducing Geobacteraceae also contain large numbers of MCPs as well as redundant sets of chemotaxis genes that are thought to play multiple roles in how these organisms sense and metabolize iron (Tran et al., 2008).

### Pilin- related genes

**Figure 9:**
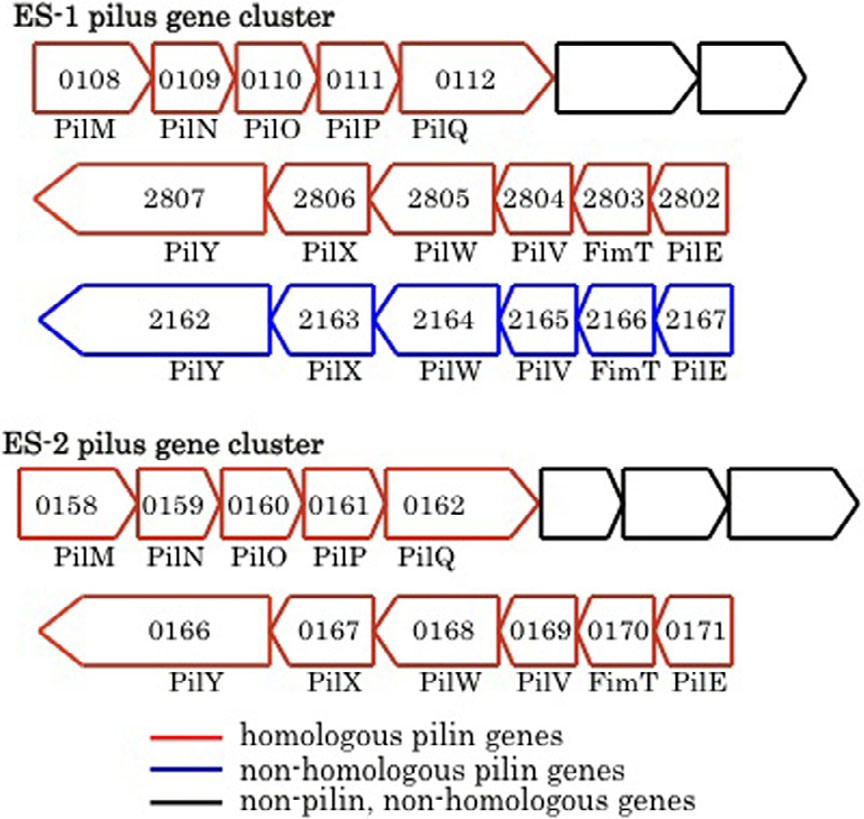
**Major groups of pilin-related genes found in ES-1 and ES-2**. The numbers inside the arrows are the IMG locus tags only for the respective genes that are homologs of pilin genes.

Pili are proteinaceous extracellular filaments that are attached as hair-like projections to the cell surface. They are found in a wide variety of bacteria and archaea, and have diverse functions that include: colonization and attachment to surfaces (including to other bacteria), secretion of exoproteins, as well as twitching motility, which allows bacteria to “crawl” along surfaces (Giltner et al., 2012). Pili contain a central pilin protein and suite of other proteins involved in processing and anchoring the structures in the membrane (Pelicic, 2008; Giltner et al., 2012). Both ES-1 and ES-2 have the genetic capacity to produce Type IVa pili. Both encode a PilMNOPQ inner membrane complex that is immediately upstream of three additional conserved genes found in this gene cluster (Pelicic, 2008), and immediately downstream of a penicillin binding protein that is also conserved (Figure 9). A second cluster of six pilin genes, *pilM/fimT/pilVWXY*, is found in both organisms. The chromosomal arrangement of the gene clusters is different between ES-1 and ES-2. In ES-2, both pilin gene clusters are adjacent to one another, but transcribed in opposite directions (Figure 9) while in ES-1, the two gene clusters are in different regions of the chromosome. Furthermore, ES-1 contains a second *pilM/fimT/pilVWXY* gene cluster that is not present in ES-2. This cluster is only weakly paralogous to the cognate gene cluster in ES-1, and is most homologous to pilin genes in *Cupriavirdis spp*. and *Ralstonia spp.* (in the Burkholderiales), Figure 9.

Both ES-1 and ES-2 contain homologous gene clusters to the MSH genes that encode another Type IVa pilus structure (Taylor and Marsh, 1999). These two gene clusters are well-conserved between the two organisms, but have undergone a significant gene rearrangement compared to the homologous gene clusters in *Pseudoalteromonas tunicata* and *Vibrio cholera* (data not shown). In *P. tunicata*, it is proposed that this set of pilin genes allows for attachment to eukaryotic algal cells (Dalisay et al., 2006); however their function in the FeOB is unknown. In addition, both ES-1 and ES-2 contain PilT (Slit_1759 and Galf_2682) and PilU (Slit_1760 and Galf_1683) that encode for pili known to be involved in twitching motility (Jarrell and McBride, 2008).

The role of pili in ES-1 and ES-2 maybe multifaceted. They could help in colonizing surfaces as a way to maintain spatial position within gradients of Fe(II) and O_2_, or promote cell–cell contact. A motility mechanism like twitching could also help the cells escape encrustation from Fe-oxides that are deposited around the cells as a result of their metabolism. In *Geobacter* spp. type IVa pili have been shown to be conductive via associated cytochromes, and play a role in extracellular electron transfer during anaerobic respiration coupled to Fe-reduction or transfer of electrons to the solid surface of an anode (Lovley, 2011). The pili-related proteins, including the primary pilin protein formed by the FeOB, are only weakly homologous to the pili proteins formed by *Geobacter*. At present it is not known if they could play any role in energy conservation from Fe-oxidation.

### Nitrogen fixation (nif) genes

*Sideroxydans* possesses three clusters of nif genes. These have the potential to encode for an FeMo type nitrogenase (White et al., 2012). The first cluster (Slit_0832 – Slit_0837) includes *nifB* and *nifQ* that are involved in biosynthesis of the iron-molybdenum cofactor in the active site of nitrogenase. The second cluster (Slit_0881 – Slit_0888) includes the *nifH, nifD*, and *nifK* genes that encode the major subunits of nitrogenase, as well as *nifT* (aka *fixT*), a short polypeptide thought to be involved in biosynthesis (Dixon and Kahn, 2004). The third cluster (Slit_0903 – Slit_0910) contains genes involved in synthesis of Fe-Mo cofactor in active nitrogenase and includes *nifE, nifN*, and *nifX*. Immediately adjacent to this gene cluster are five genes (Slit_0895 – Slit_0899) that are involved in molybdenum transport via the molybdate ABC transporter. While ES-1 is capable of growth in medium with no added N-source; its ability to fix N_2_ or express nitrogenase genes under N-limited conditions has not been tested. *Gallionella* ES-2 does not possess nitrogen fixation genes. The capacity to fix N_2_ would provide ES-1 with the ability to occupy niches depleted in N and provide a competitive advantage over organisms like ES-2 that cannot fix N_2_.

### Oxygen defense

A potentially significant problem for bacteria growing in an oxygenated environment with high Fe(II) concentrations is that the reaction of hydrogen peroxide with Fe(II) (often referred to as Fenton chemistry) can produce highly reactive oxygen species (Imlay, 2008). The production of catalase and superoxide dismutase (SOD) are common defense mechanisms microbes employ to prevent production of reactive oxygen species. *Sideroxydans* ES-1 has a single catalase gene (Slit_0713). ES-2 has a heme-peroxidase (Galf_1411) that can presumably act as either a catalase or peroxidase. In addition ES-2 has a di-haem cytochrome c peroxidase (Galf_1894) and a second di-haem peroxidase (Galf_2287). None of these genes have homologs in ES-1. Both strains have a single manganese/iron type SOD gene (Galf_2442; Slit_2312) that share high homology to one another. Many aerobic bacteria have multiple forms of SOD, so it is perhaps a little surprising that these FeOB seem to have a fairly minimal defense against toxic oxygen species. As observed above, it is also possible the abundance of hemerythrins and truncated hemoglobins could help reduce or control intracellular O_2_ concentrations.

### Metal sensitivity

Environments that have the high Fe(II) concentrations required to support the growth of FeOB, may also have relatively high concentrations of other metals and metalloids, including those that may be toxic. *Gallionella* ES-2 has a suite of mercury resistance genes, *merTPF* (Galf_1897-99) and *merA* (Galf_1900), indicating it is resistant to mercury (Barkay et al., 2003). The mercury resistance genes are not present in ES-1. ES-2 also has a cluster of five genes involved in arsenic resistance *arsRCDA* and the arsenical resistance protein ACR3 (Galf_2398 – Galf_2394; Rosen, 2002). ES-1 has *arsR* (Slit_0995), an *arsC* homolog (Slit_0996), and an ACR3 (Slit_0997) homolog, but lacks *arsA* and *arsD*. In terms of metal efflux capacity, ES-2 has 8 genes listed as members of the CzcA family of heavy metal efflux pumps. These are members of the cation diffusion facilitator (CDF) family of proteins that are responsible for removal of toxic metals like cadmium, cobalt, silver, zinc, and copper from the cytoplasm (Haney et al., 2005). In addition, ES-2 has five gene products related to acriflavin resistance genes, these are broad substrate efflux systems for the removal of antibiotics, detergents, and other potentially deleterious small organic molecules. All of these genes (both CDFs and acriflavin resistance) are associated with genes for the energy-dependent RND family of efflux transporters (Haney et al., 2005). In ES-1 there are only two *czcA* genes and two acriflavin resistance genes. Thus, it appears that *Gallionella* ES-2 is better equipped to deal with metals and other environmental toxicants in its environment, which would provide it with an advantage in waters with higher overall concentrations of metals, metalloids, or organic compounds that are hazardous to the cell.

### Polysaccharide synthesis

Neither *Sideroxydans* ES-1 or *Gallionella* ES-2 produce unique extracellular structures, such as sheaths or stalks that are often associated with neutrophilic FeOB, and are thought to help prevent the cells from becoming encrusted in Fe-oxyhydroxides. In order to help prevent encrustation, it is speculated that these cells may produce an uncoordinated exopolymer, most likely composed of a polysaccharide (Emerson and Moyer, 1997; Chan et al., 2009). Analysis of the two genomes reveals that in ES-2 there are two large clusters of genes that may be involved in polysaccharide synthesis and exopolymer production. One cluster has 41 contiguous genes (Galf_2851 – Galf_2817) that includes 9 glycosyl transferases and 6 epimerase/dehydratases and two genes annotated as homologs of polysaccharide biosynthesis genes (CapD; Galf_2833 and Galf_2823). Another cluster of 26 genes (Galf_1266 – Galf_1292) have the potential to be involved in exopolymer synthesis. This includes a polysaccharide export gene (Galf_1266), 6 glycosyl transferases, as well as a sulfotransferase (Galf_1285) suggesting the possibility for substituated polysaccharides. By comparison, ES-1 has one large cluster of 26 contiguous genes that appear to be involved in exopolymer synthesis (Slit_2879 – Slit_2904). There are 8 glycosyl transferase genes, at least 3 epimerase/dehydratase genes, and 2 polysaccharide biosynthesis genes in addition to other genes consistent with polysaccharide production. Interestingly, despite encoding for genes that may share functional overlap, the overall gene homology between the ES-1 and ES-2 gene clusters is low. ES-2 and ES-1 have other gene clusters/pathways involved in biosynthesis of lipopolysaccharide (LPS) and peptidoglycan, so it seems likely that these gene clusters may be involved in EPS production rather than production of cell wall structural glycosides.

In addition to the EPS gene clusters described above, *Gallionella* ES-2 has at least 15 genes that are associated with cellulose production that are not present in ES-1. The cellulose genes are organized into two separate clusters. One cluster, starting with Galf_0480 has seven genes identified as involved in cellulose biosynthesis, and also includes two genes (Galf_0486 and Galf_0487) that are homologs of *algJ* and *algF*, genes involved in alginate biosynthesis. A second cluster (Galf_2424 – Galf_2435) has 10 genes involved in cellulose biosynthesis; however one of these (Galf_2432), an 1179aa cellulose synthase gene is interrupted by a transposon that could disrupt the functionality of cellulose production. Direct testing of cellulose production by ES-2 has not been done.

### Secretion and transporters

The *Sideroxydans* ES-1 genome encodes a type II secretion system (Douzi et al., 2012). The first three genes of this 15 gene cluster (Slit_0506 – Slit_0489), starting with a TonB-like gene share homology and gene order with three genes in ES-2 that encode for an ABC-type transporter; the remaining genes encode for type II secretion proteins CDEFGHIJMN. ES-2 does not possess these type II secretion homologs. *Nitrospira multiformis* has a cognate set of genes that, on average, share the greatest homology with the genes in ES-1. Neither ES-1 nor ES-2 have evidence of Type III, IV, V, or VI secretion systems based on analysis of KEGG pathways.

Both organisms have the iron transport genes *feoA* (Slit_2291 and Galf_2202) and *feoB* (Slit_2292 and Galf_2203) (Kammler et al., 2003). FeoA is a small soluble SH3-domain protein, probably located in cytosol, while FeoB is a large inner membrane bound protein thought to act as a permease for Fe(II) (Cartron et al., 2006). Homologs of FeoC, a transcriptional repressor, are not found in either strain; however both strains do possess the Fur-type iron regulatory genes. ES-1 has one Fur family gene (Slit_2531) and ES-2 has three Fur genes (Galf_0862, Galf_2181, and Galf_2385) that are all paralogs.

ABC transporters are present in both ES-1 and ES-2 for sulfonate/nitrate/taurine, phosphate, branched-chain amino acids, and nickel. ES-2 has an ABC transport systems for sulfate and phosphonate that are not present in ES-1, and ES-1 has transporters for molybdate and spermidine/putrescine that are not present in ES-2. Neither organism has ABC transporters identified for any types of sugars, consistent with their inability to utilize carbohydrates for growth.

### Genes for phage and genetic recombination

**Figure 10:**
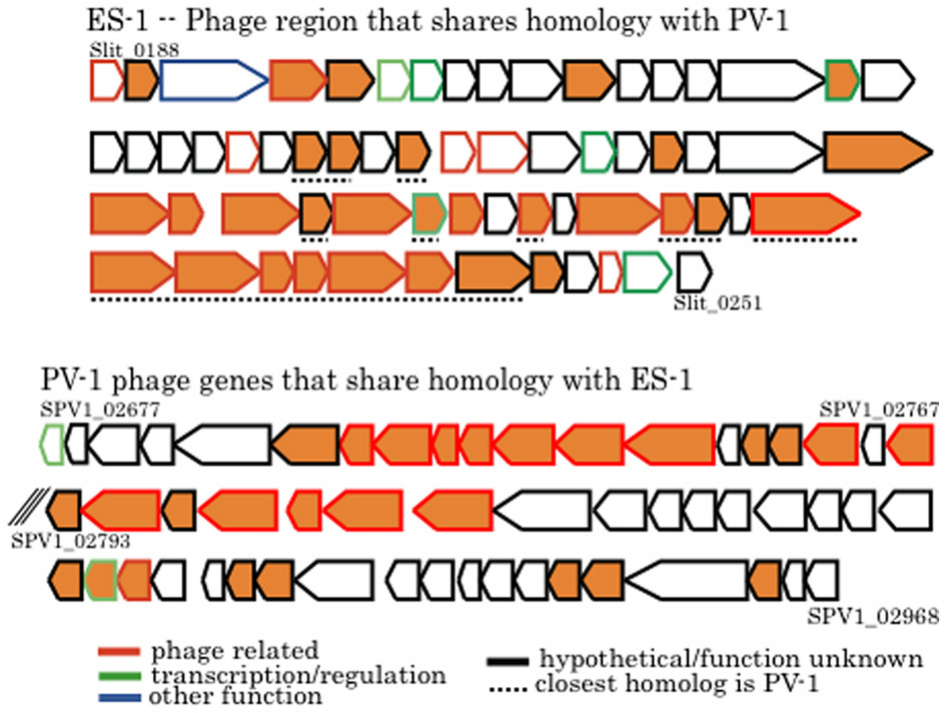
**Comparison of phage genes between *Sideroxydans* ES-1 and *M. ferrooxydans* PV-1**. The line outlining the arrow is color coded to designate putative gene function as denoted. All orange colored genes share homology between the two organisms. Numbers denote IMG gene loci; the back slashes in the PV-1 gene cluster indicate a break between two contigs.

There are two large clusters of phage genes within the chromosome of *Sideroxydans* ES-1 that presumably encode for a prophage. The first cluster (Slit_0188 – Slit_0249; 39.7 kb) has a putative phage repressor gene followed by 63 genes all transcribed in the same direction; 37 are either hypotheticals or proteins of unknown function. This cluster ends with a Mu-like prophage protein coupled with a DNA methyltransferase. Remarkably, within this cluster are 28 genes that share homology with genes in a putative prophage in *M. ferrooxydans* PV-1 (Figure 10). Sixteen of these genes are hypotheticals, or are of unknown function, and 12 are identified as phage related, most often belonging to the broad host-range Mu family of phage. The overall relatedness, both by homology and gene order, suggests that it is possible that phage-mediated gene exchange between *Mariprofundus* and *Sideroxydans* occurred at some point in the past. It is unlikely that a freshwater Fe-oxidizer from Michigan and a marine Fe-oxidizer from the central Pacific have been in close proximity in the recent past. Nonetheless, a recent study looking at the distribution of marine and freshwater Fe-oxidizers along a salinity gradient did find that *Sideroxydans*-related freshwater FeOB did overlap with members of the Zetaproteobacteria that include *M. ferrooxydans*, suggesting ancestors of these organisms could have overlapped in the past (McBeth et al., 2013).

The 2nd cluster of phage-related genes in ES-1 (Slit_1888 – Slit_1969; 54.3 kb) begins with a hypothetical, adjacent to a 16S–23S rRNA gene cluster, and has 82 genes, 67 of which are hypothetical or of unknown function. None of these phage genes have homologs with either PV-1 or ES-2, nor do they share identity with the Mu phage. ES-2 appears to have one putative prophage with 22 genes (Galf_2020 – Galf_2041; 18.9 kb) composed mostly of hypotheticals as well as several phage-related genes. None of these genes share significant identity with ES-1 or PV-1, nor do they appear to be in the phage Mu family. Neither of the two strains have identified CRISPR sequences.

The ES-2 genome has 21 genes listed as either integrase family proteins or phage integrases, and 25 transposase genes, in contrast the ES-1 genome has only three integrases and three transposases. ES-2 has a cluster of seven genes (Galf_0939 – Galf_0945) that include the *tra* genes encoding, TraF, TraI, TraU, TrbC, and TraN that are involved in synthesis of the sex pilus; however there is no evidence that ES-2 contains a conjugative plasmid. ES-1 does not possess any cognate *tra* genes. Together these results suggest there is greater plasticity in ES-2 genome for genetic exchange and recombination events than in ES-1.

## Summary of genomics

**Figure 11:**
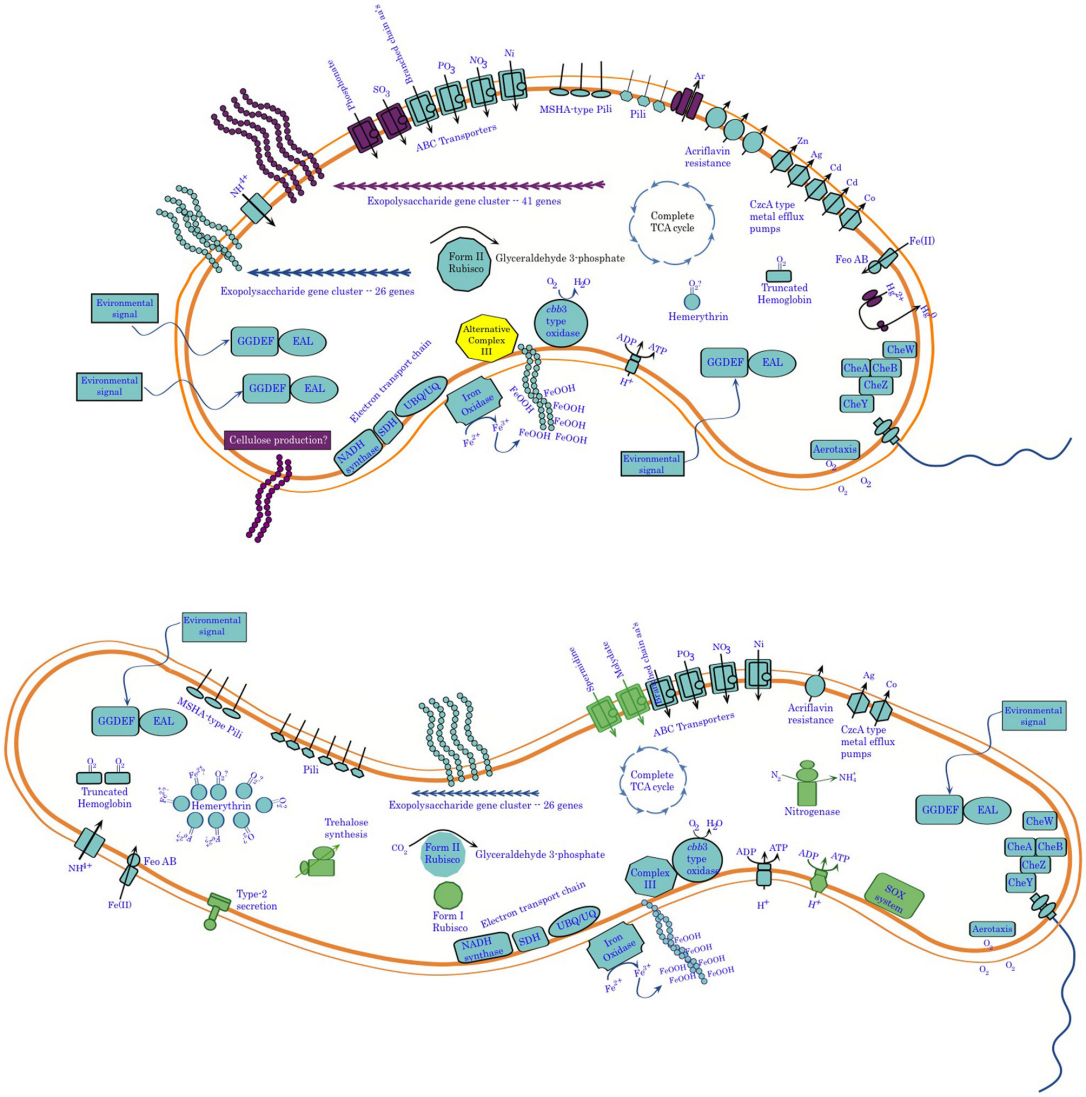
**Summary of genomic analysis of *Sideroxydans* ES-1 and *Gallionella* ES-2**. Features shown in purple in ES-2 are for functions only found in ES-2, while functions shown in green in ES-1 are not found in ES-2. In some cases, e.g., metal efflux systems, the number of features is representative of the relative abundance of the genes in the two organisms.

Comparative genomics of these two organisms confirms their overall phylogenetic relatedness and reveals functional similarities, in terms of both physiology and behavior (Figure 11). This work provides further clues for the activity of cytochrome systems being involved in conservation of energy from Fe(II) oxidation by these organisms. It also confirms the unlikelihood of there being a universally conserved set of proteins involved in Fe-oxidation among lithotrophic Fe-oxidizing bacteria. It also points up important differences between these two organisms in terms of potential niche separation, and raises intriguing questions about possible mechanisms they may use to oxidize Fe(II). Important differences include the capacity of *Sideroxydans* ES-1 to grow on thiosulfate, and to fix nitrogen. These physiological adaptations would give it an advantage over *Gallionella* ES-2 under N-limiting conditions, or in the presence of reduced S-compounds. On the other hand, ES-2 appears to be more tolerant of potentially toxic metals, which may be relatively common in high iron environments. It also is capable of producing a more varied array of exopolymers than ES-1, although the function of these is unknown they could confer advantages in preventing the cells from encrustation in Fe-oxides, or help it establish and maintain position within a gradient. Perhaps related to this is the even greater number of sensory genes that ES-2 has compared to ES-1. ES-2 may be more restricted to low O_2_ conditions than ES-1 based on its possessing fewer hemerythrins and globins, as well as the lack of a Form I type RubisCO. Nonetheless, both of these organisms are uniquely adapted for growth on Fe(II) in freshwater redoxicline habitats.

### Taxonomic descriptions

While *Sideroxydans* ES-1 and *Gallionella* ES-2 share a common metabolism in Fe-oxidation, and were isolated from the same habitat (Emerson and Moyer, 1997), the comparative analysis presented here indicates they are very different organisms that warrant separate genus and species designations. In addition to the characteristics described above that differentiate them, they are distinguished by morphology; *Sideroxydans* ES-1 is a thin, often vibrio shaped cell, while *Gallionella* ES-2 is a thicker and shorter, bean-shaped cell. Interestingly, although both organisms have gram-negative cell walls when viewed by TEM (Emerson and Moyer, 1997), they both stained gram positive when treated by conventional gram reagents following removal of Fe-oxides by treatment with either hydroxylamine or oxalic acid. To confirm this a fluorescent dye method (Mason et al., 1998) was employed on live cells (both log and stationary phase) with the same gram-positive result. The results of FAME analysis for the two strains are included in Table 4, where primary differences are in the relative abundances of the 16:0 and 18:1 w9c fatty acids.

**Table 4:** **Comparison of FAME profiles for *Sideroxydans* ES-1, and *Gallionella* ES-2**.

**Fatty acid**	**ES-1^T^a^^**	**ES-2^T^**
10:0 3OH	5.6%	3.5%
12:0	4.0	0
14:0	3.2	3.2
16:0	30.8	15
18:1 w9c	5.0	11.4
18:1 w7c	4.2	0
18:0	7.1	11.7
15:0 ISO 2OH/16:1w7c^b^	32.6	55.2

a Fatty acids that comprised <3% of total are not shown; total shown = 92.5%.

b The FAME analysis could not distinguish between these two fatty acids; they are reported together as “summed in feature 3”.

The comparative phylogenetic analysis presented here lend further support to the proposal that these two organisms and related lithotrophic FeOB belong in a novel order of the Betaproteobacteria, the Gallionellales (Weiss et al., 2007). A summary of the primary taxonomic characteristics of both strains is compared in Table 5 along with the other isolates of FeOB that belong to the Gallionellales.

**Table 5:** **Characteristics of Fe-oxidizers in the Gallionellales^a^**.

	***Sideroxydans* ES-1**	***Gallionella* ES-2**	***Sideroxydans* R-1**	***S. paludicola***	***G. ferruginea***
Isolation source:	Groundwater	Groundwater	Wetland	Rhizosphere	Groundwater
Geographic location:	Michigan, USA	Michigan, USA	Delaware, USA	Virginia, USA	Sweden
Morphology:	Helical rod	Curved rod	Curved rod	Helical rod	Curved rod
Biogenic oxide type:	Particulate	Particulate	Stalk	Particulate	Stalk
Cell diameter:	0.32 μm	0.73 μm	0.35 μm	0.42 μm	
Motile:	Yes	Yes		Yes	Yes
Microaerophilic:	Yes	Yes	Yes	Yes	Yes
Substrate utilization:					
Fe(II)	Yes	Yes	Yes	Yes	Yes
Thiosulfate	Yes	No	No	No	No
Other lithotrophy	No	No	No	No	No
Organic matter	No	No	No	No	Yes^b^
Fe(II) doubling time	8	12	15	15.8	10
Growth Temperature:										
6°C	No	Yes	No	No	Yes
30°C	Yes	No	Yes	Yes	No
pH optimum	6.0–6.5	6.0-6.5	5.6–6.1	6.0–6.5	Unknown
pH range	5.5–7.0	5.5–7.0	5.6–7.0	4.5–7.0	5.0–6.5
Reference	This work	This work	Krepski et al., 2012	Weiss et al., 2007	Hallbeck et al., 1993

a This table only includes isolates separated by ≥3% DNA sequence divergence in the SSU rRNA gene.

b Based on uptake of ^14^C-labled glucose (Hallbeck and Pedersen, 1991).

### Description of *Sideroxydans lithotrophicus* sp. nov.

*Sideroxydans lithotrophicus* (li.tho.tro'phi.cus. Gr. n. *lithos* stone; Gr. adj. *trophikos* consuming; Gr. masc. adj. *lithotrophicus* one that feeds on inorganic substrates).

This organism was isolated from an iron-mat formed in Fe(II)-containing groundwater in Michigan. The cell morphology is that of a short to medium length curved rod with a diameter of 0.2–0.3 μm that exhibits a gram-negative cell-wall by TEM, but stains gram-positive. Ferrous iron is the preferred growth substrate for this organism, although growth was also observed on thiosulfate as sole electron donor; no growth was observed on H_2_, or NH_3_, and heterotrophic growth was not observed. During growth particulate iron oxyhydroxides of no determinate shape are precipitated; the cells are associated with these oxides and are often only visible by epifluorescence microscopy. The pH range for growth is between 5.5 and 7.5 with an optimum between pH 6.0 and 6.5. The temperature range for growth was between 10°C and 35°C; the optimal growth temperature is 30°C. The cells are oxidase and catalase negative based on spot tests. The major fatty acids are 10:0 3OH, 16:0, 18:1 w9c, 18:0. The G+C content was 57.5%, and the genome size is 3.0 Mb. The type strain ES-1^T^ (=ATCC 700298^T^; JCM 14762; DSMZ 22444; NCMA B100) was isolated from Michigan groundwater.

### Description of *Gallionella capsiferriformans* sp. nov.

(cap.si.ferri.for.mans L. n. *capsa*, a box, and in bacteriology a capsule; L. n. *ferrum*, iron; L. part. adj. *formans*, forming, building; N.L. part. adj. *capsiferriformans*, an organism that forms a capsular-like precipitate of Fe-oxide).

This organism was isolated from an iron-mat formed in Fe(II)-containing groundwater in Michigan. Cells are curved or bean-shaped (~0.8 μm in diameter) and exhibit a gram-negative cell wall by TEM, but stain gram-positive. The cells are motile. The only confirmed energy source is ferrous iron; no growth was observed on reduced S species, H_2_, or NH_3_. Heterotrophic growth was not observed. During growth particulate iron oxyhydroxides of no determinate shape are precipitated; the cells are typically associated with these oxides and are often only visible by epifluorescence microscopy. This distinguishes this species from *Gallionella ferruginea* which forms a distinctive helical-shaped stalk. Growth on Fe(II) is O_2_-dependent and the cells are microaerophilic and appear to be aerotactic. The pH range for growth is between 5.5 and 7.5 with an optimum between pH 6.0 and 6.5. The temperature range for growth was between 4°C and 30°C. The major fatty acids are 16:0, 18:1 w7c, and 18:0. The G+C content was 52.7%, and the genome size is 3.0 Mb. The type strain for the species is ES-2^T^ (=ATCC 700299^T^; JCM 14763; DSMZ 22445; NCMA B101).

### Conflict of interest statement

The authors declare that the research was conducted in the absence of any commercial or financial relationships that could be construed as a potential conflict of interest.
